# A Unique Case of Pseudomonas aeruginosa-Associated Diarrhea in a Long-Term Hospitalized Adult Patient

**DOI:** 10.7759/cureus.39978

**Published:** 2023-06-05

**Authors:** Shikha Mishra, Baldeep Mann, Charizza Besmanos, Nadia Raza, Arash Heidari

**Affiliations:** 1 Internal Medicine, University of California, Los Angeles (UCLA) - Kern Medical, Bakersfield, USA; 2 Internal Medicine, University of California, San Francisco, Fresno, USA; 3 Endocrinology, University of Utah Health, Salt Lake City, USA; 4 Infectious Diseases, University of California, Los Angeles (UCLA) - Kern Medical, Bakersfield, USA

**Keywords:** c.diff, pseudomonas aeruginosa, gastroenteritis, colitis, pseudomonas

## Abstract

A 53-year-old Caucasian man with a history of alcohol use disorder, hypertension, and hypothyroidism presented with a myxedema coma requiring intubation. He had a complicated hospital course with ventilator-associated pneumonia with MRSA, sepsis with candida, and abdominal compartment syndrome requiring decompressive laparotomy. The patient slowly recovered during 43 days of hospitalization. During the intensive care unit (ICU) stay, a flexi-seal rectal tube was placed due to fecal incontinence. After being moved to a regular medicine unit, he started having loose watery stools with leukocytosis and neutrophilia. Clostridium difficile (C. diff.) colitis was suspected, and he was placed on oral vancomycin empirically. His stool test for C. diff. came back negative, and his rectal tube was subsequently removed. Imaging did not show any abscess, perforated viscus, or fistula formations. His stool culture grew a heavy colony of Pseudomonas aeruginosa (P. aeruginosa). Vancomycin was stopped, and he was started on oral ciprofloxacin 750 mg twice a day with complete resolution of his diarrhea and leukocytosis.

## Introduction

Hospital-acquired acute gastroenteritis (AGE) is a major growing concern in the hospital setting in the United States. The etiology of this entity can be infectious or noninfectious. *Pseudomonas aeruginosa (P. aeruginosa),* which is usually part of the normal flora in some conditions, can lead to invasive infection [1]. Isolation of this organism in stool culture is a rare occurrence [1]. The diarrhea caused by *P. aeruginosa* has been described mainly in the pediatric population, sometimes known as Shanghai fever [2]. Immunocompromising conditions such as malignancies, neutropenia, and admission to long-term care and the ICU are known risk factors in adults [3]. Here, we describe a case of *P. aeruginosa* associated with diarrhea in a long-term hospitalized patient with a rectal tube.

The abstract was presented as a poster at the Western Medical Research Conference (AFMR) on January 20-22, 2022, and Southern San Joaquin Valley Research Forum on May 26, 2022.

## Case presentation

The patient was a 53-year-old Caucasian male with a history of alcohol abuse, hypothyroidism not on medications, and hypertension who presented to the emergency department with abdominal pain, headaches, and altered mental status with a Glasgow Coma Score (GCS) of 3. The patient was admitted for myxedema coma requiring intubation for airway protection. The patient stayed at the hospital for a total of 43 days, which included 31 days in the ICU, with three separate times being intubated. The patient was found to have abdominal compartment syndrome requiring decompressive laparotomy. Biopsy from the exploratory laparotomy showed fibrous obliteration of the appendiceal lumen with focal noncaseating granuloma formation, leading to an appendectomy.

Regarding this case study, the important part of the hospital course is that after being transferred to the internal medicine ward on Day 28, he continued to have a rising white blood cell (WBC) count. The laboratory work is tabulated in Table 1.

**Table 1:** Laboratory workup on day 28 when the patient had diarrhea symptoms

Test	Value	Normal range
WBC	14.3 x10^3/mcL	4.5-11 x10^3/mcL
Hemoglobin	10.5 g/dL	13.2-17.4 g/dL
Sodium	147 mmol/L	136-145 mmol/L
Chloride	124 mmol/L	98-107 mmol/L
Bicarbonate	13 mmol/L	21-34 mmol/L
Blood urea nitrogen	58 mg/dL	7-18 mg/dL
Creatinine	2.29 mg/dL	0.67-1.17 mg/dL
Magnesium	2.6 mg/dL	1.8-2.4 mg/dL
Phosphorus	6.5 mg/dL	2.5-4.9 mg/dL

The patient was afebrile with no specific signs of pain. The patient was very agitated throughout the day. The patient had a rectal tube and a urinary condom catheter. The patient produced adequate urine and stools daily, but the stool from the rectal tube appeared loose. The initial concern was antibiotic-associated diarrhea and the antibiotics were stopped, as he had completed the course. The patient received nutrition via nasogastric tube due to the high risk of aspiration pneumonia. Based on leukocytosis, diarrhea, and the patient’s extended stay in the hospital, *C. diff.* infection was suspected and oral vancomycin was started. Stool culture results showed no *C. diff. *growth but was positive for* P. aeruginosa* (>100,00 cfu/ml). Further sensitivity analysis showed that the bacteria were susceptible to ciprofloxacin, gentamicin, meropenem, and tobramycin. The patient was started on ciprofloxacin 750 mg twice a day for seven days. Given the patient’s risk factors,* C. diff.* was ruled out twice through toxin polymerase chain reaction (PCR). Patient electrolyte disruption was treated with fluid correction and firm nutrition control. The patient slowly advanced to a soft diet, displayed less agitation, and regained strength. Loose stool resolved, and he began to make formed stools. The patient was then discharged to a nursing care facility to complete his recovery.

## Discussion

AGE is an emerging problem that has gained significant attention recently. It often leads to fluid, electrolyte, and nutritional deficits, which increase morbidity, mortality, length of hospitalization, and cost of healthcare. Interestingly, nosocomial AGE may also affect patients by decreasing tolerance to necessary treatments, such as antibiotics, enteral nutrition, immunosuppressants, or chemotherapies, thus decreasing the effectiveness of cancer treatment and increasing rejection rate and graft loss in transplant patients [4]. Nosocomial AGE can be caused by infectious or noninfectious agents like medications (antibiotics, antineoplastic agents, immunosuppressants, laxatives, and medications containing sorbitol or other carbohydrates) and enteral feeding [4]. The major infectious organisms that cause nosocomial AGE after antibiotic usage are *C. diff., Klebsiella oxytoca, Clostridium perfringens, *and *Staphylococcus aureus* (especially Methicillin-resistant *Staphylococcus aureus*) [4].

Due to its high prevalence in nosocomial AGE, C. diff. should be ruled out in all patients with new onset of diarrhea in the hospital regardless of antibiotic exposure. C. diff. diagnosis is established by screening with either glutamate dehydrogenase and toxin antigen followed by toxin gene PCR or toxin itself (enzyme immunoassay) used as confirmatory tests. Selective anaerobic culture and cell culture cytotoxicity assay may be used despite limited resources and time consumption [5]. Pre-existing conditions (e.g., inflammatory bowel disease) and pharmacological causes should also be considered. C. diff. is the most common cause of nosocomial infectious diarrhea, but it is not the sole cause. *P. aeruginosa* is a component of the normal flora in the human gastrointestinal tract. For this pathogen to cause colitis and diarrhea, overgrowth of the pathogen must occur, and only 15% of colonized patients presented with invasive infection [1]. Finding this organism in the stool is uncommon, but most cases involve immunocompromised patients with comorbidities and prolonged antibiotic use [1].

Pseudomonas can be isolated from usually immunocompromised and occasionally immunocompetent individuals [3]. Antibiotic use plays a large role in developing *P. aeruginosa *infection in the stool [6]. There is a reported case of an elderly woman who had an episode of bloody diarrhea associated with lower abdominal pain when being treated for a urinary tract infection with antibiotics and tested positive for *P. aeruginosa* in stool culture. In this case, in contrast to other incidents, the strain showed resistance against ciprofloxacin and levofloxacin, and discontinuation of these fluoroquinolones led to the improvement of her condition [6].

*Pseudomonas* is gram-negative, oxidase-positive, motile, aerobic bacilli. There are a variety of species that are found in soil and water contaminants. The specific species that is related to human disease pathology is *P. aeruginosa* [7]. The organism can be identified via culture plates showing some common features, such as a grape-like odor, and a variety of pigments, such as pyoverdine and pyocyanin, seen on Muller-Hinton agar plate. Notable virulence factors include pili with exoenzyme S assisting with adhesion to epithelial cells and destruction of immunoglobulin G and A; exotoxin A can cause tissue necrosis; ability to create a biofilm to increase survival and a form of antibiotic resistance; and siderophores to allow for the proliferation of the bacteria [8]. These unique characteristics come together to create multiple opportunistic infections seen by *Pseudomonas*.

Infants and children are not immune from *pseudomonas*-induced diarrhea. There have been reports where diarrhea occurred due to community-acquired enteric *Pseudomonas* infection and septicemia [9]. The occurrence of a *Pseudomonas* enteric infection and sepsis can be classified as Shanghai fever. The patients that developed Shanghai fever were typically children with high C-reactive protein (CRP), hyponatremia, and hyperglycemia [2]. There has been a case report about an elderly woman who underwent bowel resection due to colon cancer and had profuse diarrhea accompanied by a low-grade fever and abdominal cramping that complicated her hospital stay. Stool culture was positive for *P. aeruginosa* diarrhea resolved within three days of starting ciprofloxacin [10].

## Conclusions

*P. aeruginosa*-associated diarrheal disease is not common in adults. It is usually associated with complicated prolonged hospitalization, administration of broad-spectrum antibiotics, and immunocompromised conditions that perhaps play a role in colonization and, eventually, infection in the right setting. The role of the rectal tube is unknown.
